# Decoding Imagined 3D Hand Movement Trajectories From EEG: Evidence to Support the Use of Mu, Beta, and Low Gamma Oscillations

**DOI:** 10.3389/fnins.2018.00130

**Published:** 2018-03-20

**Authors:** Attila Korik, Ronen Sosnik, Nazmul Siddique, Damien Coyle

**Affiliations:** ^1^Intelligent Systems Research Centre, Ulster University, Derry, United Kingdom; ^2^Hybrid BCI Lab, Holon Institute of Technology, Holon, Israel

**Keywords:** brain-computer interface, motor imagery, imagined arm movement, motion trajectory prediction, electroencephalography

## Abstract

**Objective:** To date, motion trajectory prediction (MTP) of a limb from non-invasive electroencephalography (EEG) has relied, primarily, on band-pass filtered samples of EEG potentials i.e., the potential time-series model. Most MTP studies involve decoding 2D and 3D arm movements i.e., executed arm movements. Decoding of observed or imagined 3D movements has been demonstrated with limited success and only reported in a few studies. MTP studies normally use EEG potentials filtered in the low delta (~1 Hz) band for reconstructing the trajectory of an executed or an imagined/observed movement. In contrast to MTP, multiclass classification based sensorimotor rhythm brain-computer interfaces aim to classify movements using the power spectral density of mu (8–12 Hz) and beta (12–28 Hz) bands.

**Approach:** We investigated if replacing the standard potentials time-series input with a power spectral density based bandpower time-series improves trajectory decoding accuracy of kinesthetically imagined 3D hand movement tasks (i.e., imagined 3D trajectory of the hand joint) and whether imagined 3D hand movements kinematics are encoded also in mu and beta bands. Twelve naïve subjects were asked to generate or imagine generating pointing movements with their right dominant arm to four targets distributed in 3D space in synchrony with an auditory cue (beep).

**Main results:** Using the bandpower time-series based model, the highest decoding accuracy for motor execution was observed in mu and beta bands whilst for imagined movements the low gamma (28–40 Hz) band was also observed to improve decoding accuracy for some subjects. Moreover, for both (executed and imagined) movements, the bandpower time-series model with mu, beta, and low gamma bands produced significantly higher reconstruction accuracy than the commonly used potential time-series model and delta oscillations.

**Significance:** Contrary to many studies that investigated only executed hand movements and recommend using delta oscillations for decoding directional information of a single limb joint, our findings suggest that motor kinematics for imagined movements are reflected mostly in power spectral density of mu, beta and low gamma bands, and that these bands may be most informative for decoding 3D trajectories of imagined limb movements.

## Introduction

Brain-computer interface (BCI) research and development aims to achieve movement-free communication between a user (human or animal) and an electronic device using information encoded in neural signals. A prominent application of BCIs is controlling objects in real (LaFleur et al., 2013) or virtual spaces (McFarland et al., 2010; Royer et al., 2010) using non-invasively recorded neural signals, most commonly achieved with electroencephalography (EEG).

Sensorimotor rhythm (SMR) based brain-computer interfaces (BCIs) use voluntary modulation of the sensorimotor activity during an imagined movement (motor imagery) for communication or control. Electrodes positioned centrally over sensorimotor areas contralaterally and ipsilaterally to the imagined movement are commonly used. Multiclass classification (MC) based sensorimotor rhythm BCIs enable multi-dimensional control in real or virtual spaces using a classifier trained to distinguish between the imagined movement of different limbs, commonly the left hand, right hand, feet, and/or tongue (Pfurtscheller et al., 2006). The neurophysiology controlling these limbs produces separable features, spatially and spectrally, for the majority of BCI users. Control can be trained and learned by focused kinesthetic or visual imagery of the limb movement to activate spatially distinct cortical areas or by using a self-regulatory scheme in which the user learns to modulate the sensorimotor rhythms to gain control of the movement of an object in 2- or 3-dimensions, independently (Wolpaw and McFarland, 2004; Royer et al., 2010). Multiclass classification based sensorimotor rhythm BCIs use a feature extraction and classifier approach (extracting features that maximize the inter-class variance and minimize the intra-class variance), determining a separating plane that enables allocation of features to distinct classes (Pfurtscheller et al., 1998; Coyle, 2009; Blankertz et al., 2010).

In contrast to multiclass classification based sensorimotor rhythm BCIs that aim to classify the movement/imagined movement at any time instance, motion trajectory prediction (MTP) BCIs aim to estimate the track of the spatial coordinates or velocity vectors of moving limb joints (Georgopoulos et al., 2005). To date, most MTP studies have focused on reconstructing the movement of the upper limbs (Bradberry et al., 2010; Robinson et al., 2015), lower limbs, (Presacco et al., 2011), and fingers (Paek et al., 2014). However, to realize a movement independent BCI, reconstructing the 3D trajectory of an imagined movement is necessary. Only a limited number of papers have reported trajectory decoding of imagined limb movements in 2D (Ofner and Müller-Putz, 2015) or observed limb movements in 3D space (Kim et al., 2015), with limited success.

MTP BCIs commonly use a multiple linear regression (mLR) based kinematic data estimation module with a time-series of band-pass filtered EEG potentials as input features (Bradberry et al., 2010; Presacco et al., 2011; Toda et al., 2011; Yeom et al., 2013). Other works reported achieving reasonable reconstruction of arm trajectory using a Kalman filter (Robinson et al., 2015), kernel ridge regression (KRR) (Kim et al., 2015), or partial least squares (PLS) (Ofner and Müller-Putz, 2015).

Multiclass classification based sensorimotor rhythm BCIs normally report the highest classification accuracy using the power spectral density (PSD) of mu (8–12 Hz) and beta (12–28 Hz) bands (Pfurtscheller and Aranibar, 1979; McFarland et al., 2000). The PSD in these bands is modulated during movement planning and generation (Pfurtscheller et al., 1998; Pineda et al., 2003; Wolpaw and McFarland, 2004; McFarland et al., 2008; Royer et al., 2010). This power change is referred to as event-related (de)synchronization (ERD/S) (Pfurtscheller and Aranibar, 1979), normally measured relative to a reference period prior to the movement/imagined movement event. Lateralized differences in band power enable discrimination of one imagined movement from another (Lange et al., 2015). Beta bandpower changes are believed to be directly related to the dis-inhibition of neuronal populations involved in the specification of a motor command (Brinkman et al., 2016). In contrast, the majority of MTP BCI papers, however, report the best results when a time-series of low delta (0.5–2 Hz) band-pass filtered EEG potentials is used (Waldert et al., 2008; Bradberry et al., 2010; Paek et al., 2014). It is contended that the low delta band contains information about velocity, and trajectory for discrete (step-tracking) two-dimensional movements (Mehring et al., 2003; Rickert et al., 2005). It has been suggested that the low delta band reflects a sum of local, motor, and sensory feedback signals and is not simply considered as the arrival of input to drive movement-related activity (Reidner et al., 2011).

Slow cortical potentials (SCP)—slow direct-current shifts in the EEG, originating mainly from gradual changes in the postsynaptic potential on the pyramidal cells apical dendrites in the upper cortical layer, were also found to encode movement-related information. For example, the Bereitschaftspotential (BP)—a bilateral negative direct current shift that is detectable prior to the onset of a voluntary movement (Barrett et al., 1986), has been used to classify imagined wrist movement (Gu et al., 2009). The contralateral motor potential (MP) (Deecke et al., 1969), another type of SCP that is detectable at the time of movement execution, was also found to hold movement-related information (Birbaumer et al., 1990). SCP has also been used for controlling grasp or open a neuroprosthetic hand in a closed-loop condition (Fukuma et al., 2015). As presented by Koester et al. event-related potentials (ERPs) can be obtained during movement execution involving a grasping task (Koester et al., 2016). They showed ERP components may be related to functional components of grasping according to traditional distinctions of manual actions such as planning and control phases.

As outlined above, movement-related information is not stored in the activity of a single frequency band and can be decoded from slow DC shifts and various neural oscillations. However, limb movement classification mostly relies on the PSD of mu and beta oscillations while limb movement trajectory decoding normally involves the time-series of SCP and low delta oscillations (Müller-Putz et al., 2016). In order to study whether the poor trajectory reconstruction using activity in higher frequency bands (alpha and beta) (Yeom et al., 2013; Úbeda et al., 2017) has a biological grounding or is a shortcoming of the methods used, we replaced the band-pass filtered potential time-series (PTS) input with a PSD based bandpower time-series (BTS) in a limb movement related MTP study (Korik et al., 2016a) which was subsequently extended to a pilot study involving four subjects where we investigated both decoding 3D limb movements and imagined 3D movements (Korik et al., 2016b). Surprisingly, the BTS model provided significantly higher accuracy rates in mu and beta bands than in delta band, a result which is consistent with a substantial number of sensorimotor rhythm BCI studies using multiclass classification reporting the high classification accuracy rate using PSD of mu and beta bands. Here, we present a study with improved analysis and validation procedures, comparing the performance of limb movement and imagined limb movement decoding across 12 subjects, and take a closer look at the underlying spectral and spatial characteristics of associated brain signals. The results support our earlier findings that the bandpower time series (BTS) model is a better alternative for decoding both movements and imagined movements and that it provides the highest accuracy when the PSD of mu and beta bands are used.

Imagined limb movement classification has been reported in numerous BCI studies. Motor imagery is the focus of these studies because the aim of most BCI studies is to provide a means of allowing motor disabled patients to interact with the environment. However, limb movement classification is limited in the sense that it does not allow the decoding of the limb trajectory and therefore may not provide natural control for future prosthesis. To date, there is limited evidence that 3D movement imagination decoding is feasible using EEG. The present paper shows that this objective is feasible and suggests that, with training, subjects may learn to control prostheses and/or objects in 3D space using imagined directional movement of a single limb.

## Methods

### Subjects

Twelve right-handed male subjects (aged 25–46 years) gave informed and written consent to participate in the study, which was approved by the Wolfson Medical Center Helsinki committee. All subjects were healthy without any medical or psychological illness and/or medication and had normal or corrected to normal vision (subject 10 had brain surgery 12 years prior to the study, to remove a brain tumor in the right temporal lobe, causing epilepsy). Data acquisition took place at the Hybrid BCI lab at Holon Institute of Technology (HIT), Israel.

### Experimental paradigm

**Figure 1:**
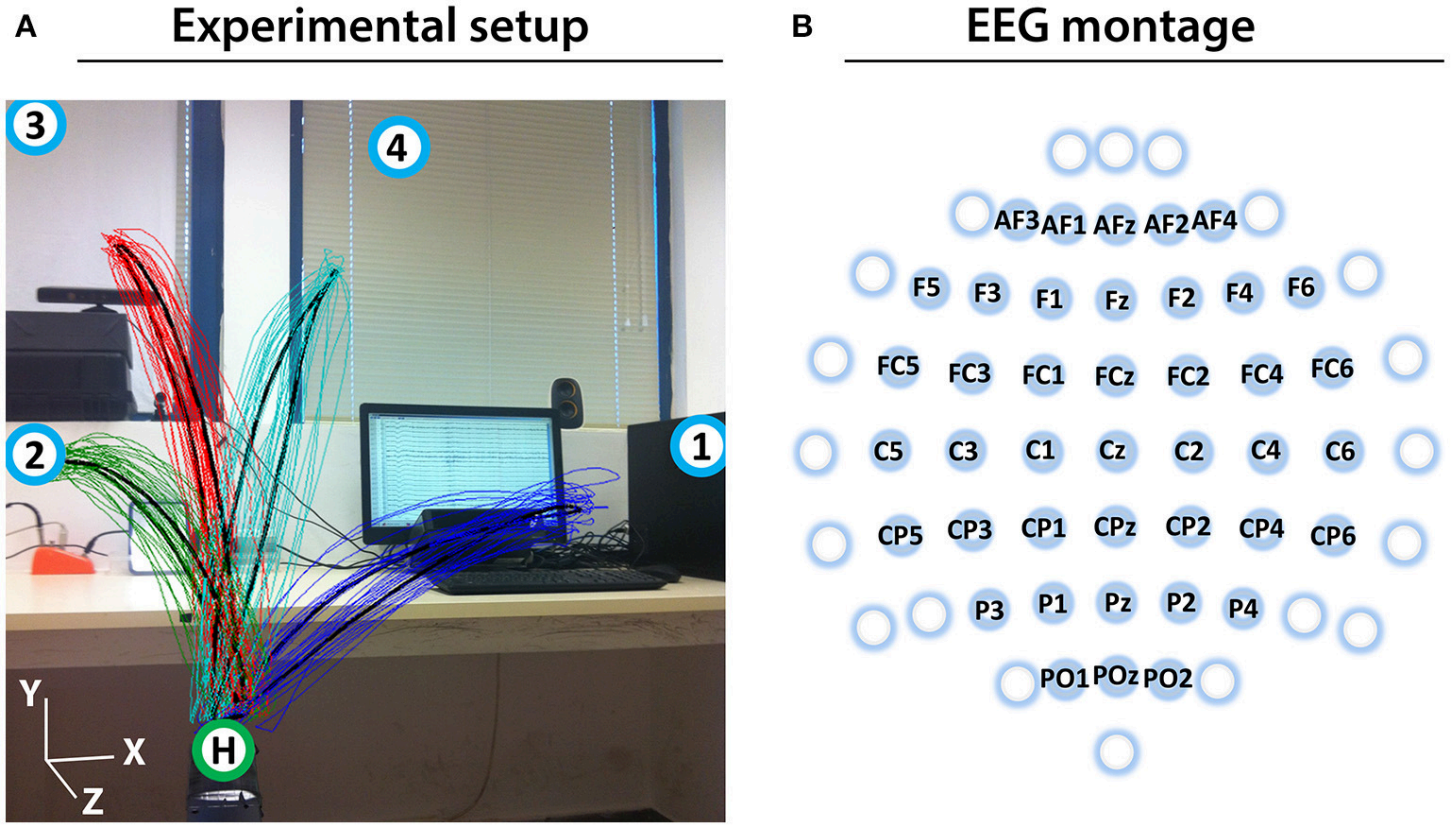
Experimental setup and EEG montage. **(A)** Experimental setup including an overlaid image of the 3D trajectory of the registered (colored thin curves) and averaged (black thick curves) arm movements between the home position (green circle with H) and four target positions (blue circles with numbers) of a representative subject (Subject 1). **(B)** The channels that were used as center points for the Laplace filtering and optimal MTP parameter selection are labeled (non-labeled channels were used only as side electrodes for the corresponding Laplace filter center positions, where required).

Prior to the experiment, the subjects were informed about the experimental protocol. Subjects were seated in an armchair positioned 1.5 m in front of a 3D Microsoft Kinect camera (Kinect, 2010; Figure 1A). The subjects were asked to look forward and maintain a constant head position, avoid teeth grinding and to minimize unnecessary movements during the experiment. They were also asked try to avoid eye blinks during the movement cycles (described below).

**Figure 2:**
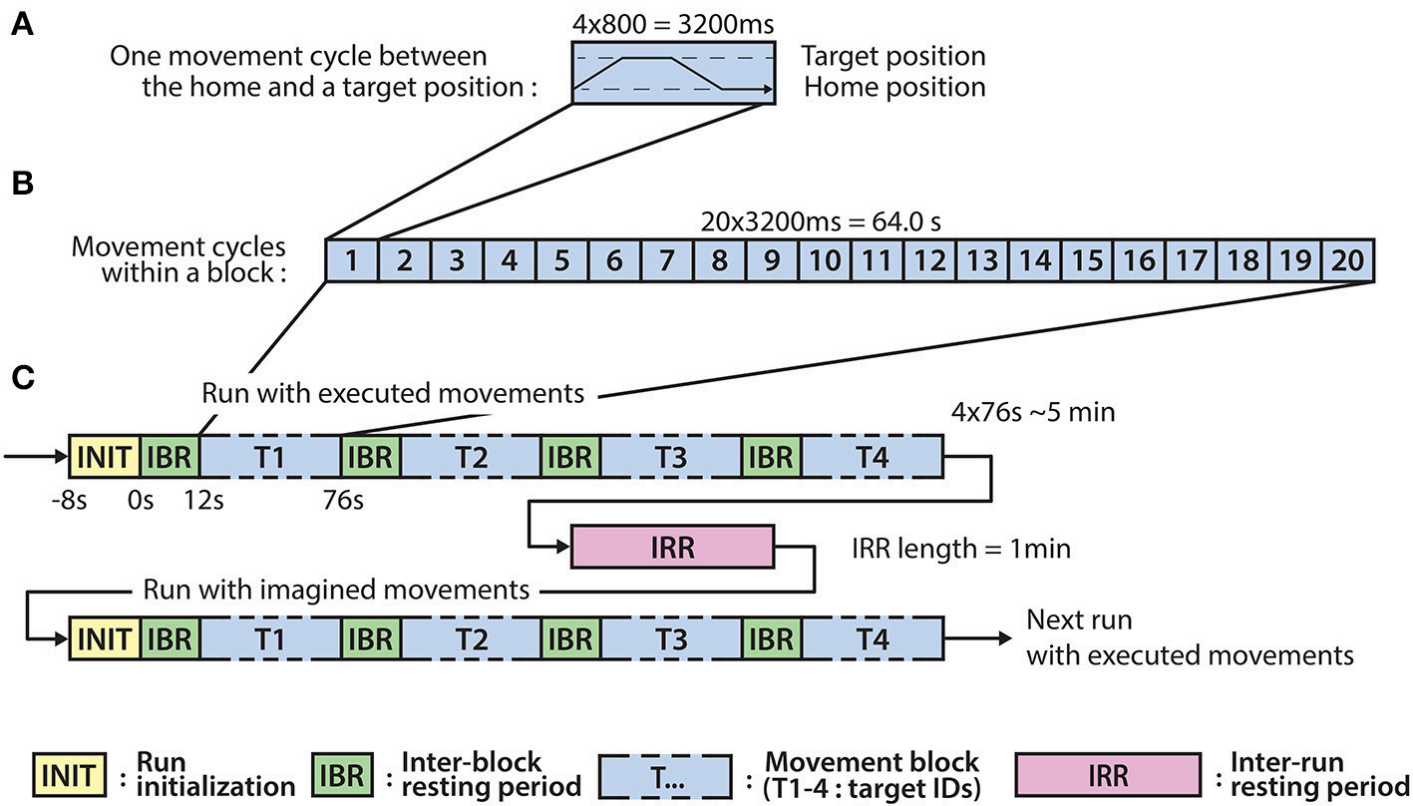
Experimental paradigm. **(A)** The timing of an executed or imagined movement cycle (depending on the run) between the home position and one of the four target positions (T1-4). **(B)** The structure of a block comprising 20 movement cycles between the home position and one of the four target positions. **(C)** The structure of the runs involving executed and imagined movements. A run involving executed movements is followed by a run involving imagined movements and the runs are separated by inter-run resting (IRR) period. A run comprises four blocks corresponding to each of the four targets (T1-4) and the blocks are separated by an inter-block resting (IBR) period.

The experiment involves eight runs, each run comprised four blocks, each block comprising twenty executed or imagined periodic arm movements between the home position and one of the four targets. For runs involving imagined arm movements, the subjects were asked to refrain from moving the hand and to kinesthetically imagine moving the arm toward the corresponding target, synchronously with an auditory cue. Each run that involved executed movements was followed by a run involving imagined movements. Inter-run resting periods lasting one minute provided an opportunity for the subject to relax, however, the subject was asked not to move or talk during the inter-run resting periods. The experimental paradigm is presented in Figure 2.

Eight seconds before commencing each run, a voice message played automatically to inform the subject about the incoming run. Twelve seconds before commencing of each block, a vocal message announced the identification number of the upcoming target (target positions were marked physically with printed labels in the environment to indicate target positions as illustrated in Figure 1A). A trial (movement cycle) comprised four epochs: a movement period between the home position and a target position was synchronized with an 800 ms length auditory cue (6 kHz tone), an 800 ms length pause at the target position without auditory cue, a movement period between the target position to the home position was synchronized with an 800 ms auditory cue (4 kHz tone), and an 800 ms pause at the home position without auditory cue. Thus, the length of a trial was 3,200 ms (Figure 2A) and the length of a movement block was 64 s and consisted of 20 similar kinematic trials between the home and one of the four target positions (i.e., repeated movement trials) (Figure 2B). The order of the targets (i.e., order of the blocks) was the same in each run (T1-4) as presented in Figure 2C (the location of the targets indicated with labels 1-4 in Figure 1A). Each run comprised four blocks, with an inter-block resting period between consecutive blocks lasting 12 s (Figure 2C), thus, the length of each run was 5 min. The runs were separated by an inter-run resting period lasting 1 min. Visual Basic software in Visual Studio was used to display and time the experimental paradigm.

### Data acquisition

EEG signals were registered with a g.HIamp80 EEG system (g.HIamp80, 2013) using 61 channels for EEG and two channels for electrooculogram (EOG) signal recording. The ground electrode was positioned on the forehead above the nose and the EEG reference electrode was positioned on the right earlobe. The EEG was amplified (gain: 20000), filtered (Butterworth, 0.5–100 Hz, 8th order), and sampled (A/D resolution: 24 Bits, sampling rate: 1,200 samples/s). The kinematic data were recorded using the 3D Microsoft Kinect camera system (Kinect, 2010), developed for the Xbox 360 game console. We used this device as it does not require markers to be placed on the joints of the arm. Kinematic data were recorded from the right hand, elbow, and shoulder at 30 frames per second (FPS). Control of an artificial or virtual arm is possible using a complex multi-joint based kinematic model. As trajectory estimation of the hand joint during 3D arm movements provides enough information to calculate all parameters of this complex arm model (Kawamura and Svinin, 2006), only hand trajectory prediction was tested. As EEG and kinematic data were registered using different data acquisition software, installed on different computers, time stamps of trigger events were stored for offline synchronizing the two signals; trigger cues from the experimental paradigm control software were simultaneously sent to the EEG data acquisition software and kinematic data acquisition software using the RS-232 serial communication protocol.

During the experiments, physical movements of the participants were recorded using a video camera which was positioned in front of the subject and the view angle was set to record the whole body apart from the legs. The recorded video was manually inspected after the experiment to confirm that no arm movements were generated during motor imagery task performance. Moreover, manual inspection of the kinematic data confirmed that the arm was idle during movement imagery epochs [i.e., did not move in any orthogonal direction more than 1.3 mm, the minimal spatial displacement (per pixel) that can be detected with the Kinect sensor (Kinect, 2010)]. Supplementary Videos 1, 2 illustrate task performance during executed and imagined movements, respectively. Although some of the subjects may have generated covert arm movements that were too small to be detected by the Kinect sensor and/or sequential co-contraction of arm muscles, it is unlikely that such movements would have a significant impact (positive or negative) on imagined 3D movement decoding accuracy.

### Preprocessing

The impedance of the EEG electrodes was measured with the g.HIamp80 EEG software package (g.HIamp80, 2013). EEG channels with impedance higher than 50 kΩ were removed from the analysis. To reduce common mode artifacts, EEG was re-referenced using a small Laplace filter (McFarland et al., 1997) centered at the 41 electrodes labeled in Figure 1B. Although all 61 electrodes were used in Laplace filtering, only 41 electrodes were denoted as Laplacian channels, completing the requirement for Laplace filtering, i.e., derived from adjacent electrodes in each (left, right, up, down) direction (e.g., electrodes of F3 were F5, F1, AF3, FC3). As the offset of the amplifiers in the EEG hardware might cause a channel specific constant baseline shift (that should be eliminated before band-pass filtering), the mean baseline value of each re-referenced channel was computed across the entire experiment and removed, separately. A 0.5–40 Hz, 8th order Butterworth filter was applied for filtering out non-relevant EEG bands. Finally, independent component analysis (ICA) was performed on the 41 preprocessed Laplacian channels using the logistic infomax ICA algorithm (Bell and Sejnowski, 1997) to remove electrooculogram (EOG) and electromyogram (EMG) artifacts (Mognon et al., 2011).

The number of removed independent components varied between four and six across subjects and the projection of the removed components (using the inverse ICA transform) was mostly over frontal cortical areas, including AF3, AF4, F5, F4, FC5, and FC6 electrodes. Here we aim to compare motion trajectory prediction (MTP) accuracy using two different approaches (i.e., EEG potentials versus PSD inputs: PTS vs. BTS), the remaining preprocessing steps differed when applying each of the two approaches.

For the band-pass filtered potential time-series based PTS model, six non-overlapped, 8th order zero-phase band-pass filters were applied separately to the ICA filtered EEG in the lower delta (0.5–2 Hz), theta (4–8 Hz), mu (8–12 Hz), lower beta (12–18 Hz), upper beta (18–28 Hz), and gamma (28–40 Hz) bands (the gap between 2 and 4 Hz is covered by the cutting edges of lower delta and theta band-pass filters). Each of the six band-pass filtered EEG datasets was re-sampled to 100 Hz.

For the PSD (bandpower) time-series based BTS model, the time-varying bandpower was calculated based on the ICA filtered EEG signals using the six non-overlapped EEG bands described above, whilst the time-varying bandpower was calculated from a 500 ms width sliding window with a 10 ms time lag between adjacent windows. This time lag was chosen to match the 100 Hz re-sampling frequency rate. The 500 ms window width is supported by the results of a pilot analysis that compared decoding accuracies obtained using four different window sizes (i.e., 50, 100, 200, and 500 ms). As the analysis (Supplementary Figure 1) did not identify significant differences using the various window-width options, that were assessed a 500 ms window-width was selected for this study, as this window-width is the shortest possible for accurate calculation of the bandpower in the lowest frequency band analyzed (i.e., in the 0.5–2 Hz low delta band). The bandpower within a time window was calculated by averaging the square values of the band-pass filtered EEG potentials as described in Equation (1).

$$\begin{array}{r} B_{fn}\left[t\right]=\frac{\sum_{m=1}^{M}{\left({S\left(m\right)}_{fn}\left[t\right]\right)}^{2}}{M} \end{array}$$

where *B*_*fn*_[*t*] is the bandpower value calculated from EEG channel *n*, using band-pass filter *f* on the same frequency ranges that are used for the potential based model (i.e., 0–2, 4–8, 8–12, 12–18, 18–28, and 28–40 Hz), within a 500 ms width time window at time *t*. *M* is the number of samples within a time window and *S*(*m*)_*fn*_[*t*] is the *m*^th^ band-pass filtered sample within the time window using the above described *f, n*, and *t* parameters.

Using a manual inspection, a high-frequency noise (>10 Hz) was detected in the kinematic data in the form of transient peaks (i.e., jitters) which did not match the curve of the joint movement but were generated by the 3D Microsoft Kinect camera system (Kinect, 2010). The moving average window involved five adjacent samples (resulting in 166 ms window width based on 30 Hz sampling rate) and the step between each window was 1 sample. Using this approach jitters were smoothed out from the movement curve and the filter did not cause significant distortion in the jitter-free curve. Data intervals involving high-level transient noise were marked during a manual inspection, and these artifactual intervals were removed from further processing along with their corresponding EEG data. Overall, less than one percent of the whole dataset was removed due to high transient noise. As it is not possible to record imagined movement related kinematic data an average of the kinematic data in the movement run prior to the corresponding imagined movement run is used to evaluate the imagined movement decoding accuracy.

### Kinematic data reconstruction

The core module of movement trajectory prediction is the kinematic data estimator block, which is dedicated to reconstructing the kinematic trajectory using an optimal time-series of the preprocessed EEG features. In the training stage, the key parameters of the estimation block are optimized. The mLR-based PTS model using time-resolved band-pass filtered potentials (the PTS model approach) proposed by Bradberry et al. (2010) is

$$\begin{array}{r} x_{i}\left[t\right]=a_{if}+\sum_{n=1}^{N}\sum_{k=0}^{L}b_{ifnk}S_{fn}\left[t-k\right]+\varepsilon \left[t\right] \end{array}$$

where *a*_*if*_ and *b*_*ifnk*_ are regression parameters representing the relationship between the input *S*_*fn*_[*t* − *k*] and output *x*_*i*_[*t*] data. *x*_*i*_[*t*] are three orthogonal components of the velocity vector where *i* represents the three spatial dimensions in the 3D coordinate system. *S*_*fn*_[*t* − *k*] is a standardized temporal difference of those EEG potentials on which band-pass filter *f* is applied at sensor *n* at time lag *k*. *N* is the number of EEG sensors, *L* is the number of time lags, and ε[t] is the residual error. The embedding dimension (i.e., the model order) is equal to the number of time lags plus one (*L*+*1*), i.e., the number of time-lagged samples that are selected from each channel for estimating kinematic data at time point *t*. The standardized difference for the PTS model is given by

$$\begin{array}{r} S_{fn}\left[t\right]=\frac{P_{fn}\left[t\right]-\mu_{P_{fn}}}{\sigma_{P_{fn}}} \end{array}$$

where *P*_*fn*_[*t*] is the value of the band-pass filtered potential based input time-series at time *t* (i.e., a potential value), μ_*P*_*fn*__ is the mean value, and σ_*P*_*fn*__ is the standard deviation of *P*_*fn*_ (μ_*P*_*fn*__ and σ_*P*_*fn*__ are calculated based on the range of time points which are involved in the corresponding training dataset, separately in the case of each training option—data separation is discussed in Section Optimal Parameter Selection and Evaluation of the Results).

The time-resolved PSD based BTS model use the same equation for mLR as described for the PTS model in Equation (2) but the standardized temporal difference *S*_*fn*_[*t* − *k*] is calculated from PSD of the specified EEG band (i.e., from bandpower values), rather than from band-pass filtered EEG potentials. As the range of the bandpower values is limited to positive values, the standardized difference is calculated differently for the BTS model compared to the PTS model (Equation 3) where the range of the input was roughly symmetric. The standardized difference for the BTS model is given by

$$\begin{array}{r} S_{fn}\left[t\right]=\frac{B_{fn}\left[t\right]}{\sigma_{B_{fn}}} \end{array}$$

where *B*_*fn*_[*t*] is the value of the PSD based input time-series at time *t* (i.e., a bandpower value) and σ_*B*_*fn*__ is the standard deviation of *B*_*fn*_.

The input-output data structures for the PTS and BTS models were prepared based on the same principles. The optimal time lag and the optimal number of lagged time points (i.e., embedding dimension minus one) was selected for both models separately, as described in the following section.

### Optimal parameter selection and evaluation of the results

**Figure 3:**
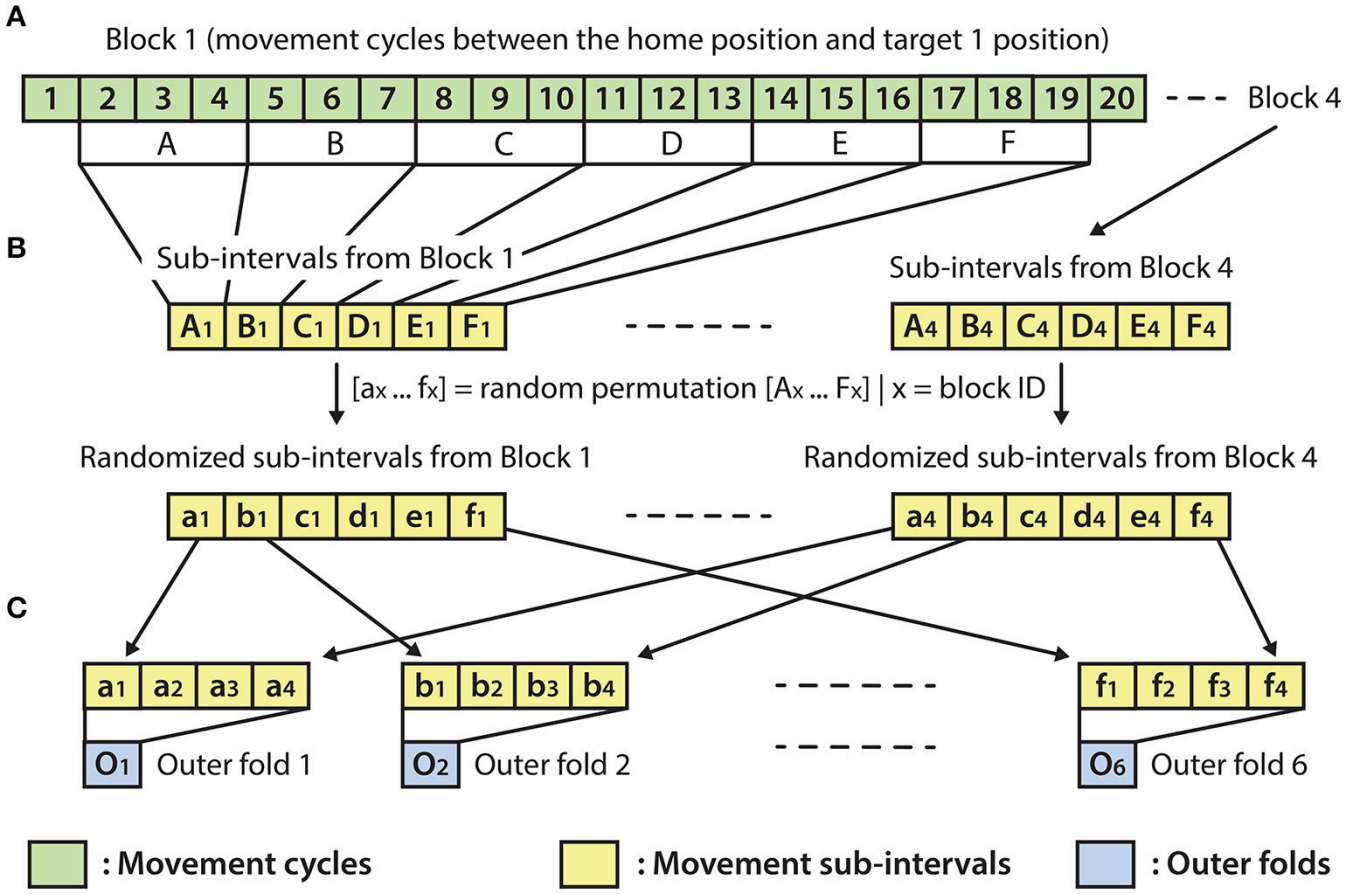
Outer fold structure**. (A)** A block with 20 movement cycles from which movement cycles 2–19 were used for the present analysis. **(B)** Re-distribution of discrete movement sub-intervals (A_x_-F_x_) in a randomized order (a_x_-f_x_), done separately for each of the four blocks. **(C)** Preparation of the outer folds involving the homogeneous distribution of each of the four movement types, respectively, to the four targets (i.e., the data for each of the six outer folds were drawn from four randomly re-distributed discrete movement intervals, with a similar length, respectively, to the four targets).

**Figure 4:**
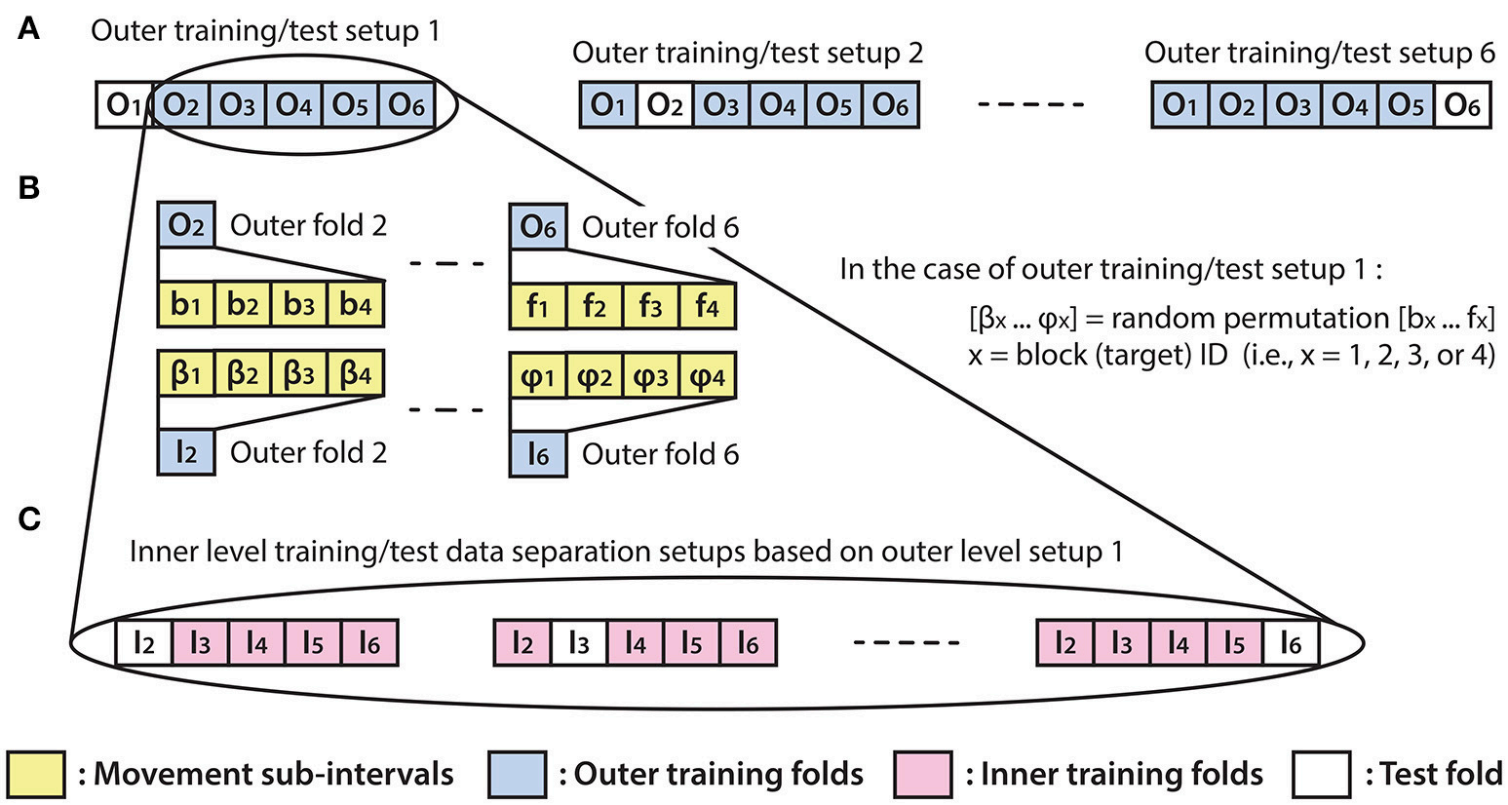
Inner fold structures. Illustration of the six training/test data separation setup options for the applied inner-outer (nested) cross-validation technique using six outer folds and five inner folds. **(A)** The outer fold setup involving six different training/test data separation options. Each of the six setup options involves five outer folds for training purposes and one outer fold for calculating the final test results. **(B)** Illustration of the outer fold training data re-distribution method for the data structure in the inner fold level of the selected outer fold. **(C)** Illustration of the inner level training/test data separation options based on the outer setup option which uses outer folds 2–6 for training and outer fold 1 for test purpose.

The optimal parameter selection and final result calculation were processed in the framework of the inner-outer (nested) cross-validation (CV) technique (Figure 4) based on the principles described in (Korik et al., 2016a) and using the structure presented in (Figure 3). As the inner-outer CV allows testing and selecting a range of parameters using an inner fold CV (Figure 4C) and calculating the final results in the outer fold CV (Figure 4A) using the optimal architecture that is selected by the inner fold CV, the final results were calculated using test data that was not used for architecture optimization. In the current analysis, six outer folds and five inner folds were used.

As the homogeneous distribution of movement dependent data intervals in the outer folds is essential for each type of movement to be weighted equivalently, the analyzed EEG-kinematic dataset was re-distributed into the six outer-folds based on a data separation method that guaranteed homogeneity (Figure 3B). Furthermore, the method described in Figure 4B provides an identical inner level data structure by mixing the movement sub-intervals within the selected outer training data folds whilst simultaneously maintaining the homogeneity in the distribution of movement dependent data intervals within the inner level data structure. Each movement sub-interval presented in Figure 4B (i.e., b1, b2, b3, b4 … f1, f2, f3, f4) covers three continuous movement cycles between the home position and one of the four target positions. Letters b, c, d, e, and f are associated with five different movement cycle triplets forming the data structure of the outer folds 2-6. Indices 1, 2, 3, and 4 are associated with the four registered blocks (four targets) within the same run. While the outer fold data structure is prepared based on movement sub-intervals a1 … f4 (as is illustrated in Figure 3), the inner fold data structure is formed by those re-distributed movement sub-intervals (i.e., in the case of the illustrated example: β1 … φ4), which are derived from the movement sub-intervals within those outer folds that are selected for training purposes at the actual outer fold setup.

The optimal time lag, embedding dimension, and most prominent frequency bands were selected in the inner level CV (Figure 4C) using a three steps approach (Table 1). Step 1: a fixed EEG montage using ten electrodes covering the sensorimotor area (i.e., FC3, FC4, C5, C3, C1, C2, C4, C6, CP3, CP4) was used to select the optimal time lag and embedding dimension. Step 2: using the optimal time lag and embedding dimension selected in the first optimization step, the importance of channels was identified by evaluating the MTP accuracy for all single channels independently and subsequently ranking channels by their importance (accuracy) and selecting a subset. Step 3: involved re-optimization of time lag and embedding dimension with the chosen subset of best channels from the second optimization step. The optimal time lag, embedding dimension, and most prominent frequency bands were selected by the inner level CV (Figure 4C) while the final results were calculated based on the outer test folds (Figure 4A), using the optimal architectures that were selected in the inner level CV.

**Table 1:** The parameter space used for optimal parameter selection.

**Parameter**	**Parameter space**	
	**PTS model**	**BTS model**
Time lag	10…200 ms	100…300 ms
Embedding dimensions	1…13 samples	
Frequency range	0.5–2 Hz, 4–8 Hz, 12–18 Hz, 18–28 Hz, 28–40 Hz	

As the accuracy of trajectory reconstruction in MTP BCIs is assessed by comparing the trajectory of the performed movement and the reconstructed limb movement (Georgopoulos et al., 2005; Bradberry et al., 2010; Yeom et al., 2013; Paek et al., 2014; Kim et al., 2015; Robinson et al., 2015), the Pearson product-moment correlation coefficient between the two descriptors was computed separately for each investigated setup and served as an accuracy metric. The optimal parameter selection was run for each model (PTS and BTS), movement type (imagined or executed), subject, run, frequency band, and velocity component (v_(x)_, v_(y)_, and v_(z)_), separately. In order to obtain the optimal EEG montage, the MTP was trained and tested using a single channel input. The test accuracy values were calculated separately for each of the 41 Laplace filtered and further preprocessed EEG channels in each of the five inner folds. Each channel was assigned an average score based on the Pearson correlation value and was ranked. The eight EEG channels that provided the highest accuracy rates were included in the EEG montage. The final results were calculated by averaging MTP accuracy across the outer folds for each subject, separately.

In order to assess the validity of the obtained trajectory reconstruction accuracy, a shuffling test was performed. For the shuffling tests, the trajectory was reconstructed using the original (i.e., non-shuffled) EEG test dataset but the order of the blocks was shuffled in the kinematic test dataset. Thus, the reconstructed trajectory in the shuffled test was compared with an incorrect target trajectory. The correlation of the reconstructed and shuffled target trajectories is expected to be low. The correlation values between shuffled and non-shuffled tests from six outer folds were compared using the Student's two-tailed *t*-test, separately for the various investigated options (i.e., model type, movement type, subjects, and bands).

In order to study the contribution of each of the Laplace filtered EEG channels to trajectory reconstruction, the average accuracy rate was computed for the 12 subjects in 6 × 5 inner folds. For subject specific topographical maps, all Laplacian channels were assessed by checking the contribution to trajectory reconstruction that is significantly higher than expected given the null hypothesis (i.e., all channels have the same contribution). To that end, for each subject and fold, the R value (correlation between neural activity and one of the coordinates) of each Laplacian channel was normalized by the highest R value and the absolute value taken (looking for a high correlation - both positive and negative). Next, for each channel, the R values from all folds of the actual subject were pooled together and a *t*-test was run (checking whether the mean of R values minus the expected value is higher than zero). As each channel was assessed, the p value was corrected for multiple comparison [*p* < 0.001, Student two-tailed *t*-test corrected for FDR (false discovery rate)]. Finally, significant R values were normalized and plotted, separately for each MTP model, movement type, subject, and frequency band, in the form of a subject specific topographical map. Next, in order to identify those cortical areas which provide a significant contribution to trajectory reconstruction across 12 subjects, the contribution of each Laplacian channel was estimated using 6 × 5 inner folds. Again, Laplace filtered channels that contributed significantly higher than expected given the null hypothesis (i.e., all channels have the same contribution) were determined [*p* < 0.001, Student two-tailed *t*-test corrected for FDR (false discovery rate)]. Finally, the R values were normalized and plotted (for channels which successfully completed the *t*-test), separately for each MTP model, movement type, and frequency band, in the form of a cross-subject topographical map.

The cross-subject averages of reconstructed trials for imagined and executed movements were calculated, separately, using the PTS and BTS models. Each average trial was calculated using twelve subjects, six outer folds, and four runs based on low delta information for the PTS model and based on mu, lower beta, upper beta, and gamma information for the BTS model. The cross-subject average of reconstructed trials and an example of single velocity trial reconstruction for imagined and executed arm movements using the PTS and BTS models are presented in the results section.

**Figure 5:**
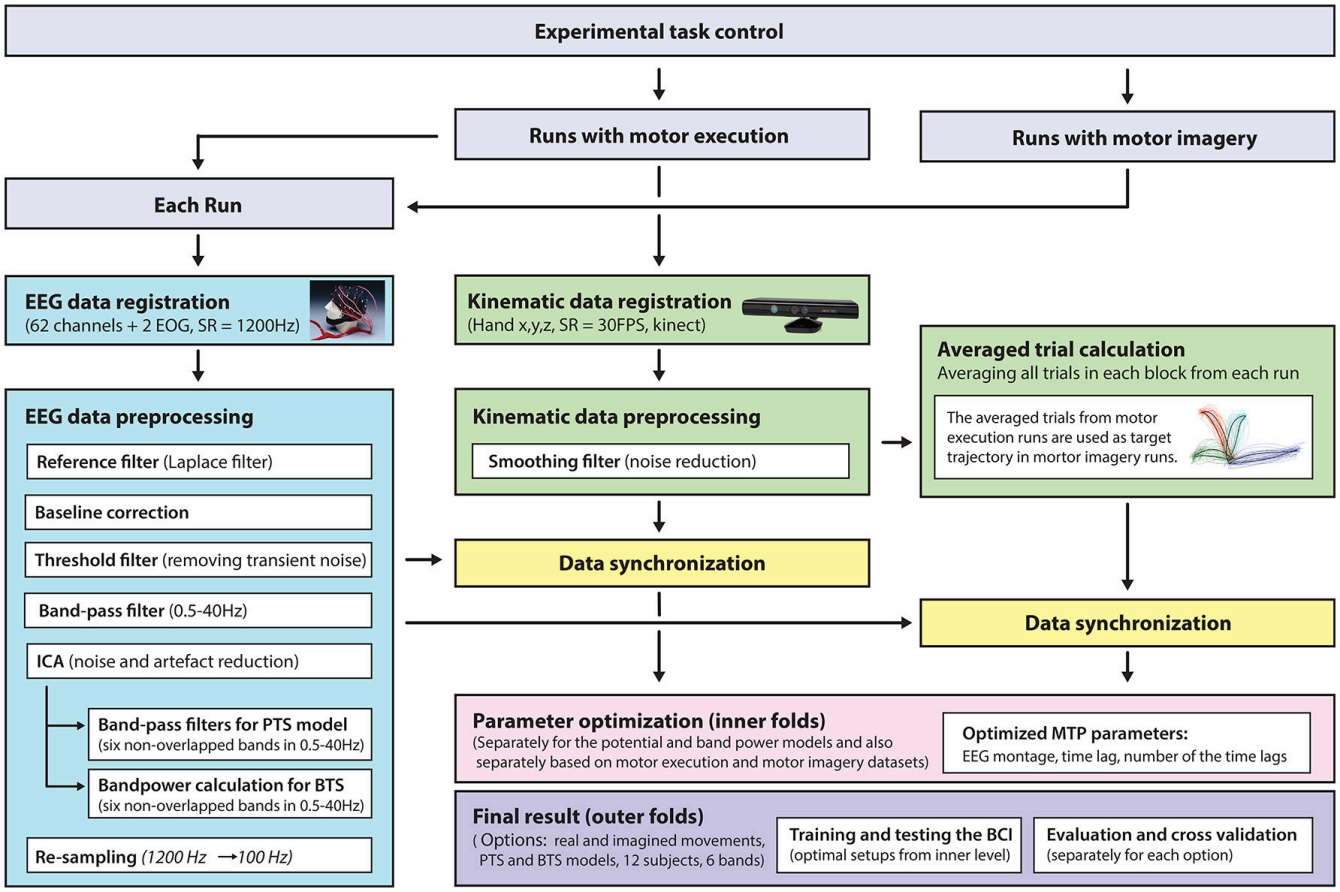
Signal processing pipeline—from data acquisition to evaluation. EEG and kinematic data were parallel registered during the task and preprocessed offline. For motor imagery runs, the target kinematic trajectories were calculated by averaging the kinematic data in the executed movement run prior to the corresponding imagined movement run. The optimal time lag, embedding dimension, and most prominent frequency bands were selected using a three-step procedure for finding optimal parameters. The parameter optimization and final results were calculated in framework of the inner-outer cross-validation technique.

A general overview of the signal processing steps and evaluation blocks is illustrated in Figure 5.

## Results

**Figure 6:**
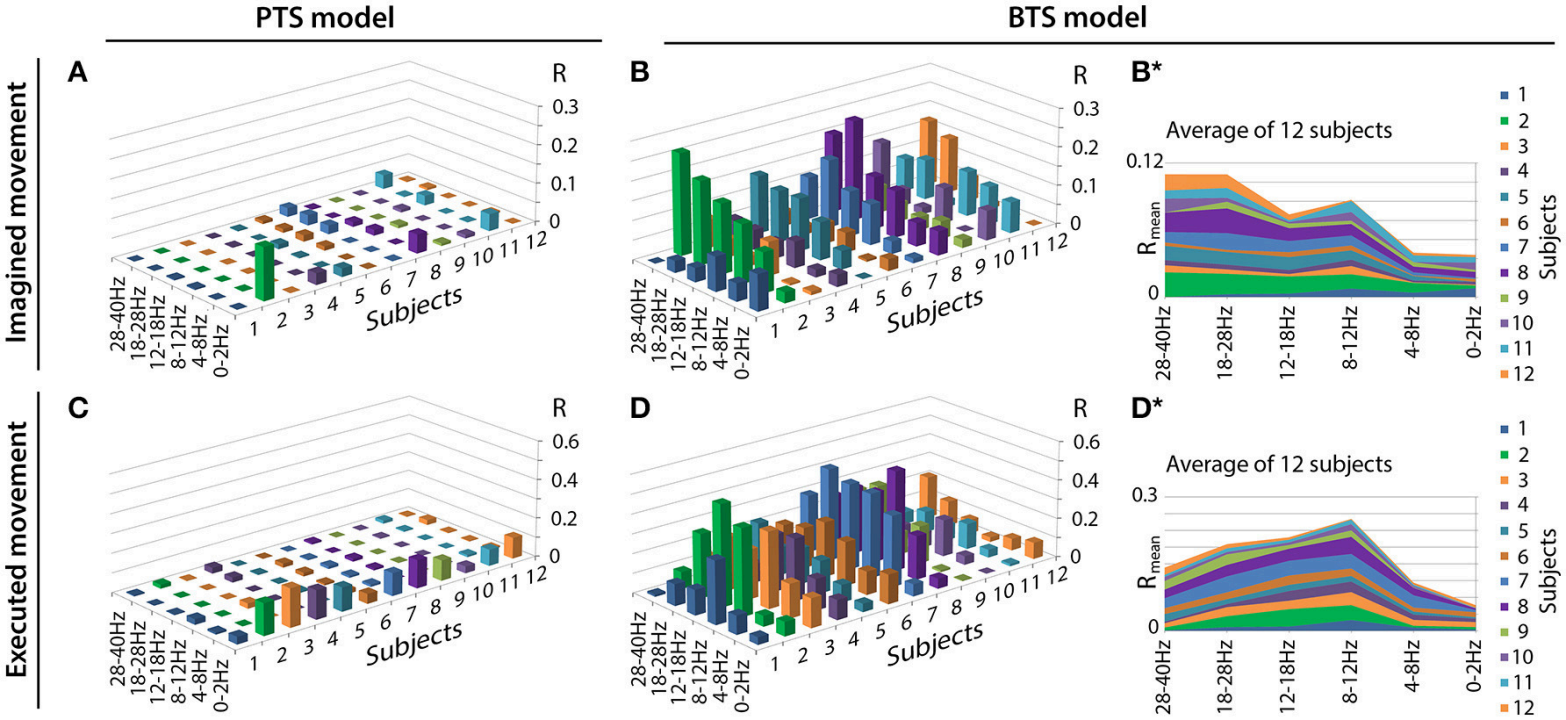
Motion trajectory prediction accuracy using different models. Each displayed accuracy value is an average value based on the results of four runs, six outer folds, and three velocity components. Decoding accuracy values of imagined arm movements are presented in **(A)** for the band-pass filtered potential based PTS model and **(B)** for the PSD based BTS model. Reconstruction accuracy of executed arm movements is presented in **(C)** for the band-pass filtered potential based PTS model and **(D)** for the PSD based BTS model. For the BTS model, the cross-subject average of the reconstruction accuracy is presented in (B^*^) for imagined and in (D^*^) for executed movements (similar comparison of cross-subject mean values for the PTS results is not presented in this Figure as panels **(A)** and **(C)** show that the low delta band (0–2 Hz) is the dominant band for each subject for the PTS model).

Figure 6 illustrates the accuracy rates obtained for executed and imagined arm movement trajectory reconstruction. In line with other MTP studies (Bradberry et al., 2010; Yeom et al., 2013; Paek et al., 2014), the accuracy of the PTS model using band-pass filtered potential time-series input was maximal when a low delta band (0.5–2Hz) band-pass filter was applied ($R_{Executed}^{PTS}\sim 0.15$) and it was very low for other frequency bands (R~0) (Figures 6A,C). In contrast to the PTS model, the BTS model using bandpower time-series enabled reconstruction of the executed movements with the highest accuracy ($R_{Executed}^{BTS}\sim 0.4$) using the time-resolved power spectral density of the mu (8–12Hz) and beta (12–28Hz) bands (Figure 6D). For imagined arm movements, the PTS model provided low accuracy for all frequency bands ($R_{Imagined}^{PTS}\sim 0$). In contrast to the PTS model, the BTS model achieved higher accuracy ($R_{Imagined}^{BTS}\sim 0.2$) in the mu (8–12Hz), beta (12–28Hz), and low gamma (28–40Hz) bands (Figure 6B) for imagined arm movements. The validity of the results was confirmed by a shuffling test as described in the Methods section. The original (non-shuffled) dataset provided significantly higher accuracy than the shuffled dataset for both models (PTS and BTS) (*p* < 0.05, Student two-tailed t-test).

**Figure 7:**
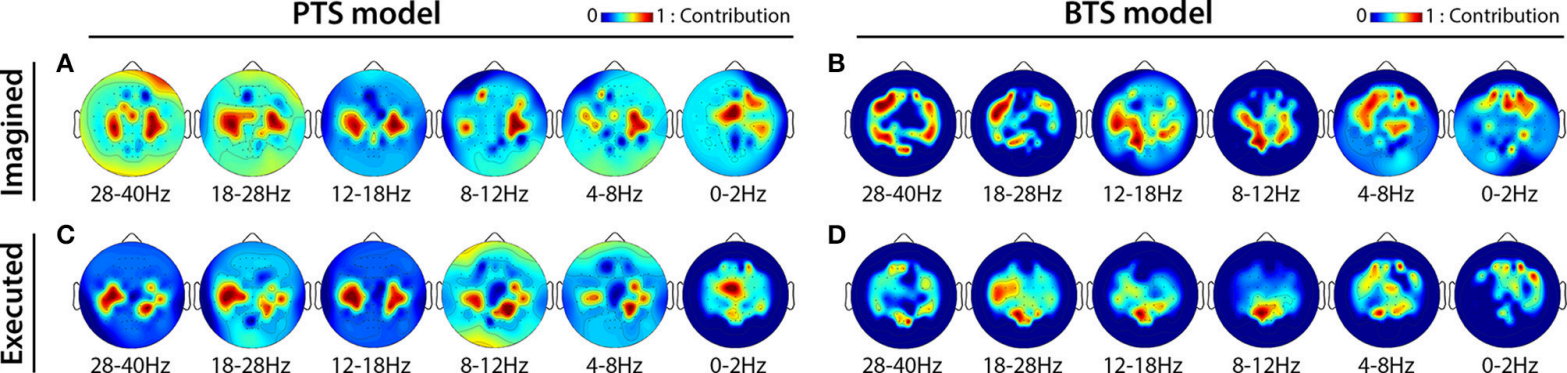
Cross-subject based topographical maps. Topographical distribution of electrode contribution to the reconstruction of arm movement trajectory. Color coding—averaged contribution of significant channels [*p* < 0.05, Student two-tailed *t*-test corrected for FDR (false discovery rate)] based on accuracy rates of single channel setups over 12 subjects indicating cortical areas providing a high contribution for trajectory decoding. **(A,C)** present topographical maps for imagined and executed arm movements using band-pass filtered potentials for the PTS model, respectively. **(B,D)** Present topographical maps for imagined and executed arm movements using PSD values for the BTS model, respectively.

**Figure 8:**
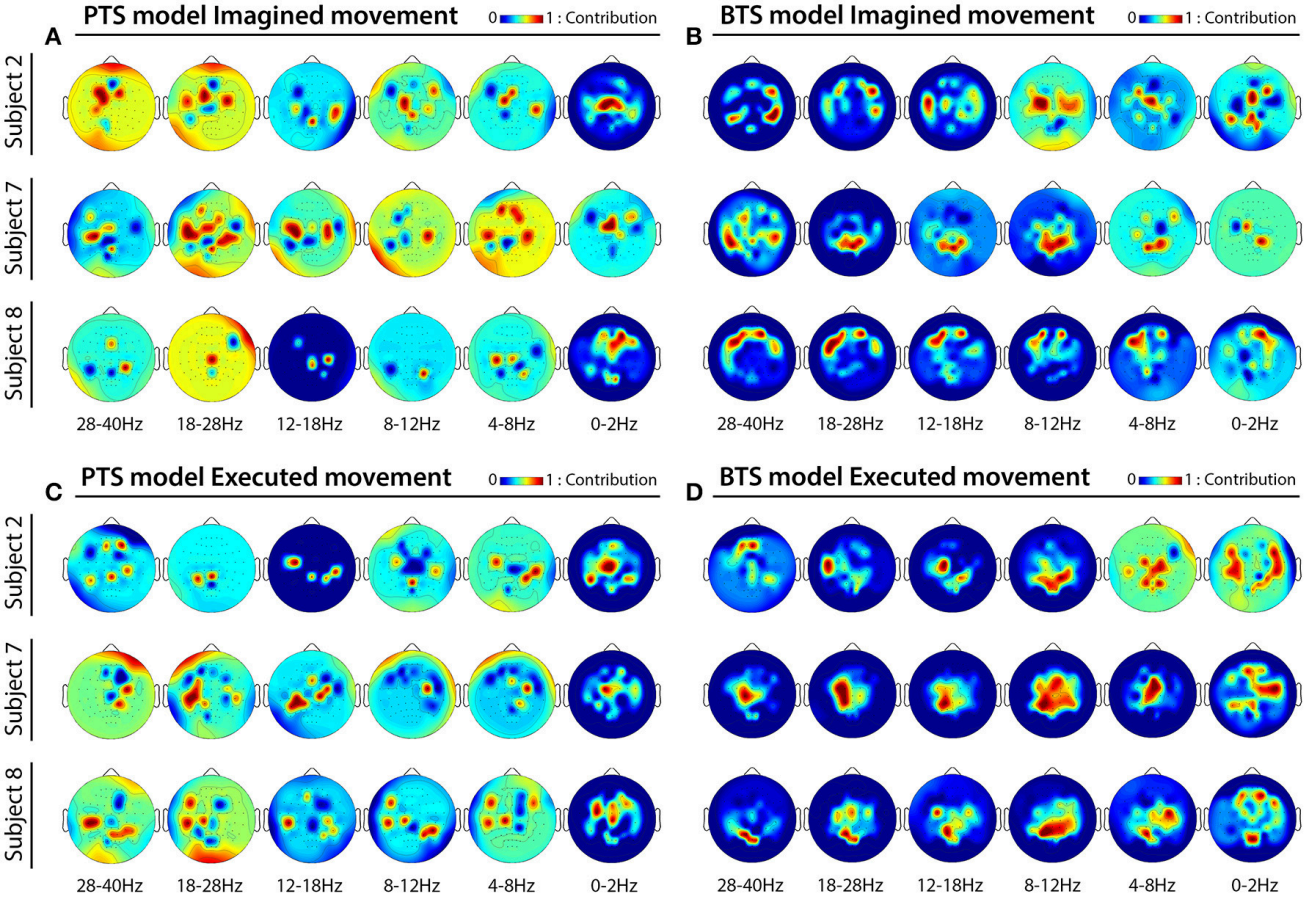
Subject specific topographical maps. Topographical distribution of electrode contribution to the reconstruction of imagined and executed arm movements for subjects 2, 7, and 8 [i.e., subjects for whom reconstruction accuracy was highest (see Figure 6)]. Color coding—contribution of significant channels [*p* < 0.05, Student two-tailed *t*-test corrected for FDR (false discovery rate)] based on accuracy rates of single channel setups in 6 × 5 inner folds indicating cortical areas providing a high contribution for trajectory decoding. **(A,C)** Present topographical maps for imagined and executed arm movements using band-pass filtered potentials for the PTS model, respectively. **(B,D)** Present topographical maps for imagined and executed arm movements using PSD values for the BTS model, respectively.

MTP accuracy values using different single channel setups were calculated (i.e., the MTP BCI was trained and tested for each analyzed EEG channel, separately) and the accuracy values were calculated for each model (PTS and BTS), subject and frequency band, separately. The topographical distribution of the electrodes' contribution to trajectory reconstruction revealed that different sets of electrodes conveyed most of the information regarding the generation of arm movements and imagined arm movements. With the PTS model, electrodes positioned over the sensorimotor cortex conveyed most of the trajectory-related information, as evident in Figures 7A,C showing group (i.e., cross-subject) topographical maps (also in Figures 8A,C single-subject topographical maps). For the BTS model during executed movements, the highest accuracy is derived from the contralateral sensorimotor and occipital cortex when the movement was decoded from one of the two most prominent (i.e., mu or beta) frequency bands, as illustrated for 8–12, 12–18, and 18–28 Hz frequency ranges in Figure 7D (and in Figure 8D). The prominent cortical areas for imagined movement using the BTS model are more diversified across different subjects, as presented in Figure 7B (and in Figure 8B). For some subjects, the most prominent electrodes were positioned over the frontal cortex (e.g., subject 8 in Figure 8B), whereas for some, occipital located electrodes conveyed most of the information (e.g., subject 7 in Figure 8B).

**Figure 9:**
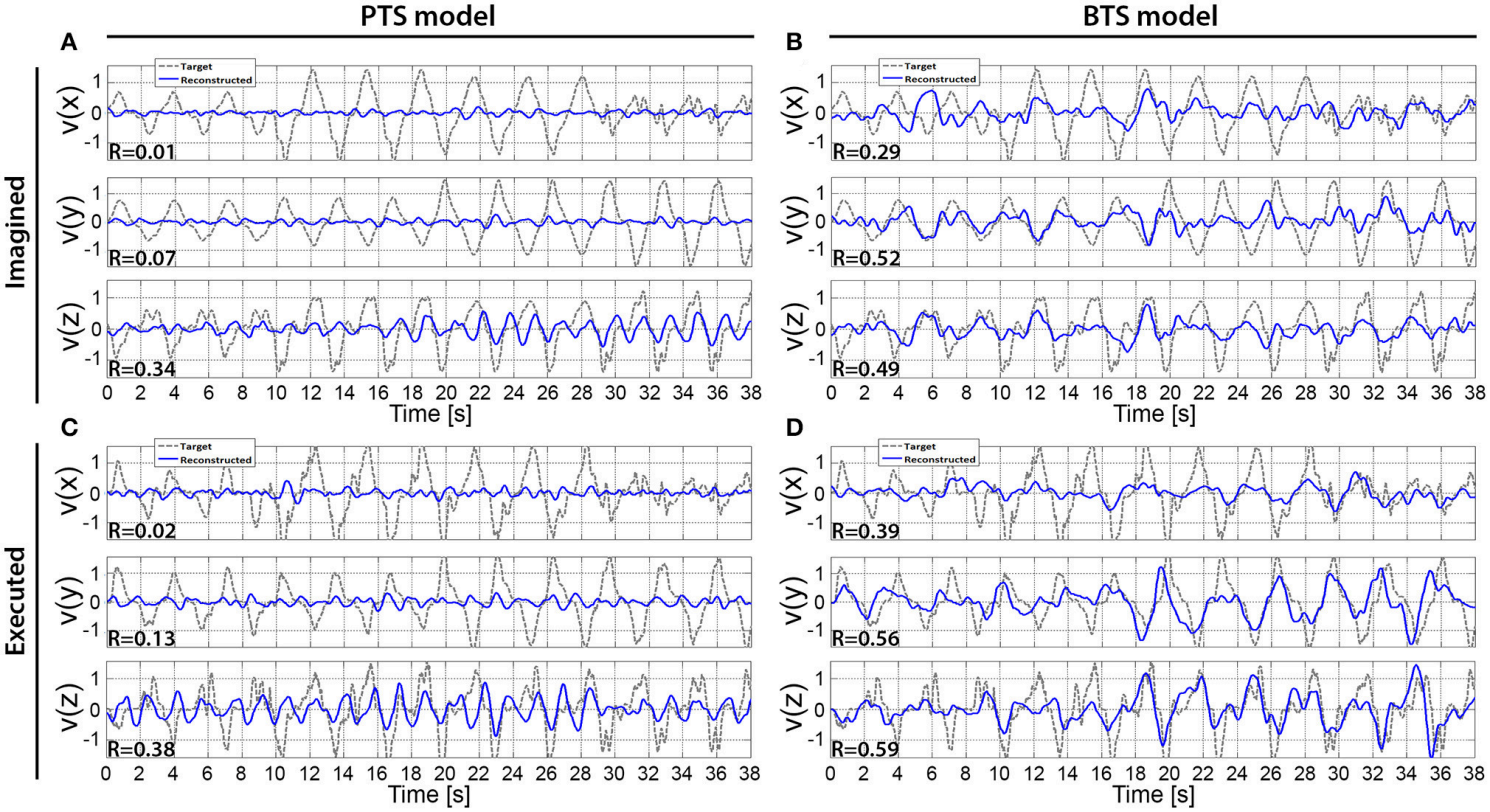
Reconstructed hand velocity vector components of single kinematic trials. V(x), v(y), and v(z) velocity vector components are matched with executed or imagined movement in horizontal, vertical, and depth directions, respectively. For the imagined movement reconstruction **(A,B)**, the target trajectory was calculated based on executed arm movement trials that were registered using the experimental protocol that was used for the run with imagined arm movements. The imagined arm movement velocity reconstruction is presented in **(A,B)** for the PTS and BTS models, respectively. The executed arm movement velocity reconstruction is presented in **(C,D)** for the PTS and BTS models, respectively. **(A,B)** are calculated based on subject 2, run 4, and outer fold 1 using the time-resolved low delta (0–2 Hz) band-pass filtered EEG for **(A)** and PSD of the low gamma (28–40 Hz) band for **(B)**, while **(C)**, and **(D)** are calculated based on subject 2, run 2, and outer fold 1 using the time-resolved low delta (0–2 Hz) band-pass filtered EEG for **(A)** and the time-resolved PSD of the low beta (12–18 Hz) band for **(D)**.

**Figure 10:**
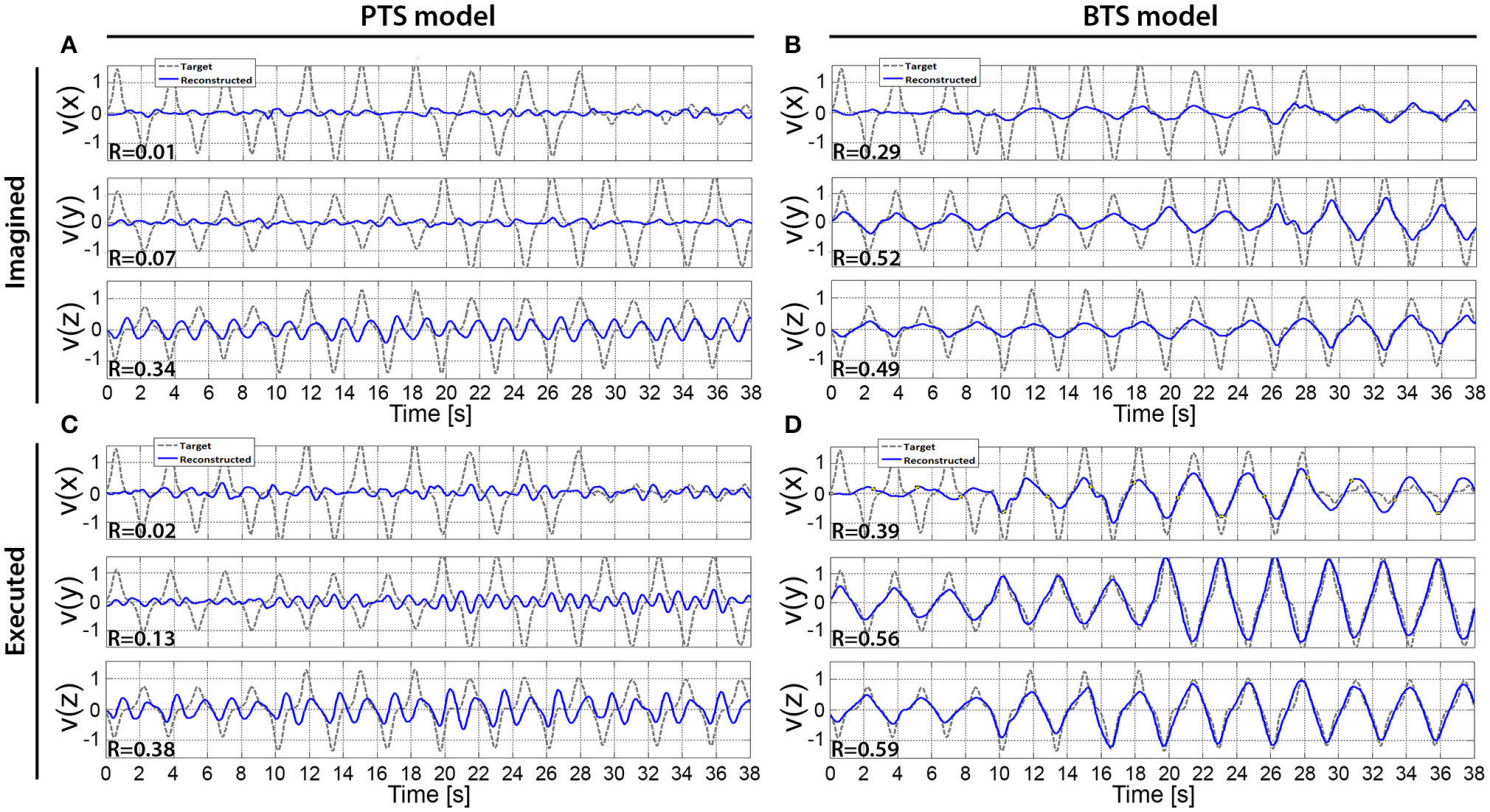
The cross-subject average of reconstructed hand velocity vector components. V(x), v(y), and v(z) velocity vector components are matching with executed or imagined movement in horizontal, vertical, and depth directions, respectively. For the imagined movement reconstruction **(A,B)**, the target trajectory was calculated based on arm movement trials. The averaged trial of the imagined arm movement velocity reconstruction is presented in **(A,B)** for the PTS and BTS models, respectively. The averaged trial of executed arm movement velocity reconstruction is presented in **(C,D)** for the PTS and BTS models, respectively. The averaged trial for the PTS model (**A,C**) involve results of time-series predictions using the time-resolved low delta (0–2 Hz) band-pass filtered EEG. The averaged trial for the BTS model **(B,D)** involve results of time-series predictions using PSD of the optimal band selected in the inner level cross-validation [i.e., mu (8–12 Hz), lower beta (12–18 Hz), upper beta (18–28 Hz), and gamma (28–40 Hz) bands].

An example of a single velocity trial reconstruction using the PTS and BTS models for movement and imagined movements is presented in Figure 9. Model, subject, and frequency bands used for Figure 9 were selected for subjects with the highest accuracies (Figure 6). The cross-subject average of reconstructed trials is presented in Figure 10.

## Discussion

### PSD of mu, beta, and low gamma oscillations for decoding imagined arm movements

Decoding the trajectory of imagined movements from EEG has been reported in only a limited number of studies. The aim of this study was to investigate if the trajectory of 3D hand movements could be decoded from EEG and determine which neural oscillations and detection methods provide maximal decoding accuracy. We evaluated the possibility of decoding imagined 3D arm movements by decoding the imagined 3D trajectory of the right (dominant) hand and its relationship with the average trajectory of 3D movements using time-resolved band-pass filtered potentials and time-resolved PSD values, in six non-overlapped EEG bands covering the 0.5–40Hz frequency range. The results of this study, which focused on direct and implicit decoding of the trajectory of the hand during kinesthetically imagined 3D arm movements (i.e., neither motor execution nor movement observation but kinesthetic motor imagination in 3D spaces), provided a clear evidence that mu, beta, and low gamma oscillations are more likely to provide better performance for MTP of imagined 3D arm movements using a power spectral density estimation approach compared with low delta oscillations using a band-pass filtered EEG potential approach.

To the best of the author's knowledge, all other 3D motion trajectory studies to date involve arm movements and not imagined arm movements and most arm motion trajectory prediction (MTP) BCIs use time-resolved band-pass filtered EEG potentials (referred to here as the PTS model) for reconstructing the 3D trajectory of the movement (Bradberry et al., 2010; Presacco et al., 2011; Paek et al., 2014). Closely related imagined/observed movement studies include (Kim et al., 2015; Ofner and Müller-Putz, 2015). Although Kim et al. decoded 3D trajectory of executed and imagined arm movements with multiple linear regression (mLR) and kernel ridge regression (KRR) methods in (Kim et al., 2015), the motor imagery task was performed in parallel with observation of a human volunteer or robot performing 3D arm movement. In Ofner and Müller-Putz (2015) the motor imagery task was synchronized with a metronome, the required imagery movement was not presented during the motor imagery task, and the task involved performing imagery of arm movement in vertical and horizontal directions of a two dimensional (2D) plane and not a complex three dimensional (3D) movement. As outlined, decoding the 3D trajectory of an imagined movement is very much understudied - both of the above cited studies (i.e., Kim et al., 2015; Ofner and Müller-Putz, 2015) used time-resolved band-pass filtered potential values from the low delta band and do not support the use of low delta band information for MTP in imagined 3D arm movement. The present and our recent study (Korik et al., 2016a) showed that trajectory reconstruction of 3D movement is possible using time-resolved PSD values (referred to here as BTS model) and mu, beta, and gamma oscillations but not delta oscillations.

For the PTS model, reasonable accuracy rate for both movement and imagined movements was achieved only in the low delta band. A mathematical evidence for explaining this observation is discussed for executed movements in Korik et al. (2016a) and summarized below. As the movement followed a characteristic period of 1.6 s (Figures 9, 10), corresponding to a 0.625 Hz frequency, it is logical to suppose that only the 0–2 Hz band will contribute to the decoding using a band-pass filtered potential time-series with a multiple linear regression based model. If the band-pass is applied to a frequency range which is significantly higher than the characteristic frequency of the movement, the samples in the potential time-series pick-up quasi-random values of the band-pass filtered EEG. In other words, a band-pass filtered potential time-series input of the mLR based PTS model represents information content of the EEG correctly if and only if the band-pass filter matches the characteristic period of the movement cycles (i.e., applied to the 0–2 Hz low delta band). For the BTS model, this issue does not exist as time evolution of bandpower values in any EEG sub-band match the above described characteristic period, therefore, the BTS model represents correctly the information content of the EEG in a wide range of different sub-bands.

However, the accuracy rate for imagined movements (Figure 6A) was much lower than observed for movements using the low delta band (Figure 6C). This result in agreement with (Babiloni et al., 2017), who observed in a reach and grasp task that the ECoG delta and theta (<8 Hz) band contain more information for movement execution than for movement observation. Our results show that although 3D trajectory reconstruction of imagined arm movements can be realized using time-resolved potentials from the low delta band, the low delta band encodes less information related to imagined arm movements compared to that observed for movement. In contrast to the PTS model, the BTS model using time-resolved PSD values for reconstructing movements (Figure 6D) and imagined movements (Figure 6B) achieved the highest accuracy rate using information from the mu, beta, and low gamma bands.

### Topographical analysis

The topographical analysis showed different results using time-resolved band-pass filtered potentials for the PTS model and time-resolved PSD values for the BTS model (Figure 7). The PTS model decoded maximal trajectory information from the sensorimotor cortex in both types of movement (i.e., imagined Figure 7A and executed Figure 7C), as expected (Paek et al., 2014; Ofner and Müller-Putz, 2015). Although using the BTS model, the sensorimotor cortex has been detected as the most important cortical area for decoding trajectory information of an executed movement (Figures 7D, 8D), for some subjects, the frontal or occipital cortical areas were also important for decoding an imagined movement (Figures 7B, 8B). Neuper et al. showed that the cortical activity during motor execution and kinesthetic motor imagery is focused in the contralateral sensorimotor area, whereas during movement observation and motor imagery the frontal and occipital areas, respectively, show higher contribution for hand movement classification (Neuper et al., 2005). It may be speculated that some subjects have used different methods (e.g., kinesthetic or visual imagery) to imagine arm movement (Dickstein and Deutsch, 2007), although explicitly asked to use motor memory during the imagined movement task. Movement-related modulation of mu and beta activity in the motor cortex is discussed in Miller et al. (2010), Miller et al. (2012), and Gwin and Ferris (2012). Halder et al. showed that the active cortical area for BCI users who achieve higher performance in a motor imagery tasks is not limited to only the sensorimotor cortex, and have found activations in the right middle frontal gyrus (Halder et al., 2011). The trajectory relevant frontal activity for an imagined arm movement (subject 8 in Figure 8B) might originate from the planned movement as planning is probably more important when motor imagery is being performed, particularly if a subject is performing motor imagery for the first time, as is the case for all subjects in this study. This observation paired with the low gamma results for imagined MTP is in line with a study by Ball et al. who reported the planned movement-related oscillations associated with the gamma activity in the frontal cortical areas (Ball et al., 2008), and a study by Thürer et al. shows increased gamma activity following retrieval of motor memory after a period of consolidation in a dynamic adaptation task (Thürer et al., 2016). Limb movement visualization is also a possible explanation for the increased MTP accuracy when occipital activity is used for imagined arm movement estimation. If a subject concentrated on motion visualization instead of performing an imagined kinesthetic task, an increase in neural activity in the visual cortex may occur (Halder et al., 2013), resulting in higher MTP accuracy using signals from occipital areas, as observed for subject 7 during imagined movement tasks (subject 7 in Figure 8B). In summary, however, our analysis could not clearly link cortical areas and cognitive strategies for the best imagined movement prediction due to the variability in the topological results across 12 subjects.

### Reconstructed trajectories

The results across twelve subjects (Figure 10) show greater estimation accuracy for both arm movements and imagined arm movements using PSD time-series of mu and beta bands compared to using the time-series of low delta band-pass filtered EEG potentials. A comparison of target and reconstructed trajectories (Figure 9 shows an example for single subject trajectories and Figure 10 presents results using cross-subject average) indicates better reconstruction accuracy in the vertical (y) and depth (z) movement directions (especially for executed movements) compared to the horizontal (x) plane. This difference might originate from the topographic distribution of the targets, as the angle between different targets, is greater measured from the view point of the home position, for the horizontal coordinates compared to the vertical or depth coordinate component. The greater angular difference in the horizontal plane may be more difficult to achieve for the subject during the motor task execution, and may impact on the results, especially in the case of the executed movement tasks. The reconstructed trajectories using cross-subject average show better fitting with the target trajectories than those obtained for the single subject trajectories (Figure 9 vs. Figure 10), suggesting that the target and reconstructed trajectories are correlated across different subjects.

### A closer look at the techniques and paradigms used

This study uses a block design paradigm involving repeated movements to one target in each block of trials. The block design paradigm, previously used in several similar studies (e.g., Ofner and Müller-Putz, 2015), involve repeated movements to each of the four targets. Block design was preferred, rather than cueing the subject before each trial to imagine a movement to any one of the four targets, as a single trial design is often more complex and cognitively challenging for the subject and is a more time consuming experiment as a cue period is required before each trial. This cue could also have confounding influence on the neural response e.g., ERP or stimulus onset effect. A block design also minimizes the risk of a subject imaging a movement to a wrong target and reduces eye movements (gazes to the target) that could impact on the signal. With a block design, it is possible that repeated movements to a single target consecutively could result in a motor kinesthetic memory that leads to the evolution of segregated, distinct neural patterns, which result in high classification accuracy (as shown in Sosnik et al., 2016). Whether this effect has an influence on motion trajectory prediction accuracy compared to that of using a random trial sequence based paradigm remains to be investigated, however, any effect on accuracy will affect both models and frequency band approaches compared in this study. Future work will explore the variations in performance for single trial vs. block design.

There is no exact trajectory to evaluate the imagined movement decoding accuracy and therefore the averaged kinematic trajectory was calculated in the movement run prior to the corresponding imagined movement run. Here we provide a justification for this. The variability of the averaged trials (calculated for the same target in each run, separately) was analyzed across four runs as follows: First, averaged trials were calculated based on the same target for the same subject and same vector component but based on different runs. Next, the cross-correlation of averaged trials corresponding to the same target, subject, and vector component was calculated for each paired-combination of the four runs, separately. The cross-correlation values obtained using twelve subjects, four targets, three vector components, and all combination of four runs were very high (the mean R value was 0.972 with a standard deviation of 0.056). This result indicates that a target-related averaged trial computed for a specific run can represent an imagined movement between the home position and the corresponding target position.

As training the model on an averaged trajectory, overlooking variability in movement kinematics, may bias decoding accuracy rate, decoding accuracy rates in runs involving executed movements were computed and compared using the following two approaches: 1. trajectory information from each individual movement trials were used as targets for each movement trial i.e., each movement trial trajectory differed based on the inconsistency of limb movement and 2. average movement trajectory for movement trials (i.e., averaged across trials in a block) was used as the training target for all movement trials i.e., each movement trial target was identical. Test results were calculated for both approaches and no significant differences in decoding accuracy (*p* > 0.05, Student two-tailed *t*-test) were found (Supplementary Figure 2). As replacing identical trials with an averaged trial in the training dataset to predict executed arm movements did not result in a significant difference in decoding accuracy, identical trials in the kinematic training data can be replaced with averaged trials, without biasing the decoding accuracy rate with respect to the variability of the kinematic training dataset. We are, therefore, confident that using the averaged trials for imagined movement prediction from the corresponding motor execution block, prior to the run involving imagined movements, does not bias the results.

Performance metrics have been investigated in the context of motion trajectory prediction based BCIs. Antelis et al. reported that a BCI employing multiple linear regression (mLR) models using periodic movements may lead to an overestimated accuracy in the low delta band if the metric is the correlation between target and reconstructed trajectories (Antelis et al., 2013). As we use correlation to compare methods and frequency band performance, it is important to emphasize that Paek et al. observed that resting state EEG yielded R-values centered at r = 0 across subjects, indicating that high R-values cannot be attained from randomly generated data and therefore correlation is an appropriate measure to use (Paek et al., 2014).

Though the decoding accuracy was higher for the BTS model using PSD values ($R_{Executed}^{BTS}\sim 0.4$) compared to the PTS model using band-pass filtered potentials ($R_{Executed}^{PTS}\sim 0.15$), the observed accuracy rates are relatively low compared to a number of studies reporting accuracies of R~0.3–0.7 for executed upper limb movement reconstruction using the standard PTS input (Bradberry et al., 2010; Liu et al., 2011). This difference could be a result of an over-sensitive ICA applied in the present study for removing muscular artifacts. For example, in some cases, an executed movement might have some influence on the signals in low-frequency bands, i.e., the executed movement might cause an effect on the electrodes as discussed in Castermans et al. (2014) for a walking task based study. Eye movement following the movement may also have an impact on non-invasive recordings. However, in Kim et al. (2015), where movement observation was combined with imagery, even though the EOG-related activity was removed using ICA the residual effect of the visual observation could have an influence on results. Kim et al. reported that electrooculographic contamination of EEG can have a significant impact on mLR approaches that use low delta band information, which can be avoided using a nonlinear model (Kim et al., 2015). In our study, the ICA was applied to remove any such distortions and as we have focused on imagined movement only, neither artifacts caused by movement of limbs to targets nor movement observation could have an influence on the observed imagined movement results.

Although 12 subjects participated in the experiment, the results should be validated with a higher number of subjects. In addition, it would be advantageous to study subjects over longer and/or more sessions to investigate performance improvement and learning in a closed loop scenario.

## Conclusion

To date, electroencephalography (EEG) based decoding accuracy of arm movement trajectories is found to be maximal using information in the low delta band. The most commonly studied EEG-based brain-computer interface (BCI) decodes directional information of an executed limb movement from a band-pass filtered potential time-series. In the present study, we replaced the time-resolved band-pass filtered EEG potential based potential time-series input with a time-resolved power spectral density based bandpower time-series. The accuracy rates of three dimensional (3D) trajectory reconstruction of the right (dominant) hand for executed and imagined arm movements were compared for both approaches in six non-overlapped bands selected within the 0.5–40 Hz frequency range. Our results not only show that the 3D trajectory of an imagined arm movement can be decoded from power spectral density of the mu, beta, and low gamma bands but the bandpower time-series based model provides higher accuracy rates in these bands compared to the potential time-series based model produces with the low delta band. The results present evidence that the power spectral density of mu, beta, and low gamma bands encode movement-related information during imagined 3D arm movement, thus, mu, beta, and low gamma bands are better candidates for imagined 3D arm movement decoding than the low delta band. The evidence presented here also corroborates the evidence from the extensive literature on classical motor imagery BCI paradigms that predominantly use mu and beta oscillations and rarely use low delta oscillations. The results support the development of BCIs which may enable physical movement independent 3D control of artificial or virtual limbs. Our current focus is on evaluating the proposed methods in a closed-loop BCI where users imagine 3D arm movements to control virtual arms in real-time. BCIs that decode imagined movements are necessary for applications that are targeted at enabling physically impaired individuals perform movement-independent communication and control in real and virtual spaces.

## Author contributions

AK: Carried out the research, computation and data analysis, and prepared the figures and draft of the manuscript; RS: Did the data collection, contributed to the topographical analysis, and revised the manuscript; NS: Reviewed and revised the manuscript and contributed to the optimal parameter selection section; DC: Supervised the research, contributed to experimental design, data and results verification, literature search and discussion of the results, and revised the manuscript.

### Conflict of interest statement

The authors declare that the research was conducted in the absence of any commercial or financial relationships that could be construed as a potential conflict of interest.
