# Supplementary material for: Decoding Imagined 3D Hand Movement Trajectories From EEG: Evidence to Support the Use of Mu, Beta, and Low Gamma Oscillations
Source: Front Neurosci. 2018 Mar 20;12:130. doi: 10.3389/fnins.2018.00130 (PMC5869206; doi:10.3389/fnins.2018.00130)
Supplement: Supplementary file 3 [file Image1.PDF]

Supplementary Material

Decoding Imagined 3D Hand Movement Trajectories from EEG: Evidence to Support the use of Mu, Beta, and low Gamma Oscillations

Attila Korik*, Ronen Sosnik, Nazmul Siddique, Damien Coyle

*** Correspondence:** Corresponding Author: korik-a@ulster.ac.uk

1 Supplementary Figures

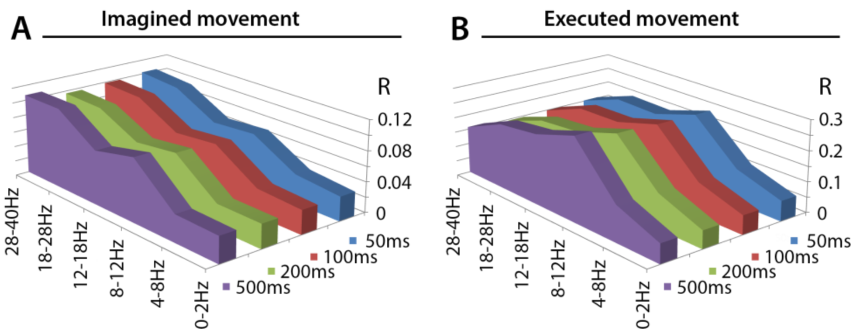

Supplementary Figure 1. Comparison of the MTP accuracy rates of the BTS model using different time window widths for bandpower calculation. The accuracy of the BTS model was compared using four different window sizes (i.e., 50ms, 100ms, 200ms, and 500ms) for the bandpower calculation. This figure is prepared by averaging test results of the twelve investigated subjects. **(A)** Results of the imagined arm movement prediction. **(B)** Results of the executed arm movement prediction. As the results show similar accuracy rates for each investigated bandpower window, a 500ms width window was selected for the main part of the present study as this window size is the shortest for calculating properly bandpower in the analyzed lowest frequency band (i.e., in the 0.5-2Hz low delta band).

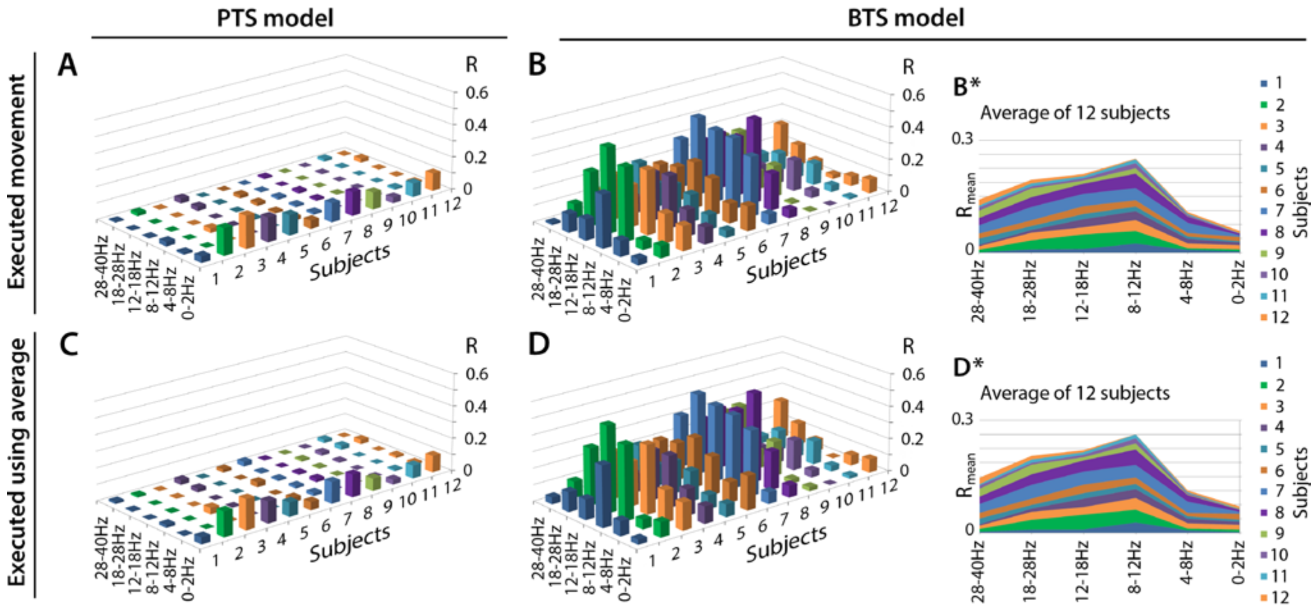

Supplementary Figure 2. Movement trajectory prediction accuracy values using identical **((A)** and **(B))** versus averaged **((C)** and **(D))** kinematic trials for training the band-pass filtered potential time-series input based PTS **((A)** and **(C))** and the power spectral density time-series input based BTS **((B)** and **(D))** models. For the BTS model, the cross-subject average of the accuracy values is displayed in **(B*)** using identical trial for training and in **(D*)** using averaged trial for training. The averaged training trials for **(C)** and **(D)** are computed using twelve similar kinematic trials that were registered in the same block corresponding to the same target. Each displayed accuracy value is an average using test result of four runs, six outer folds, and three velocity components.
